# Nailfold Capillaroscopy Changes in Patients with Idiopathic Inflammatory Myopathies

**DOI:** 10.3390/jcm13185550

**Published:** 2024-09-19

**Authors:** Milan Bogojevic, Milica Markovic Vlaisavljevic, Rifat Medjedovic, Elvira Strujic, Dragana Pravilovic Lutovac, Slavica Pavlov-Dolijanovic

**Affiliations:** 1 Department of Rheumatology, Clinical Centre of Montenegro, 81000 Podgorica, Montenegrorifat.medjedovic@gmail.com (R.M.); draganact84@yahoo.com (D.P.L.); 2 Faculty of Medicine, Institute of Rheumatology, University of Belgrade, 11000 Belgrade, Serbia; slavicapavlovdolijanovic@gmail.com

**Keywords:** idiopathic inflammatory myopathies, nailfold capillaroscopy, scleroderma pattern, dermatomyositis, polymyositis

## Abstract

**Background/Objectives:** Idiopathic inflammatory myopathies (IIMs) are rare autoimmune disorders characterized by progressive proximal muscle weakness and varying extra-muscular manifestations. The latest 2017 EULAR/ACR criteria classify them into subgroups. This study aims to evaluate the role of nailfold capillaroscopy (NFC) as a diagnostic and prognostic tool in IIMs by comparing capillaroscopic patterns across different IIM subtypes. **Methods:** We conducted an observational, cross-sectional study at the Institute of Rheumatology in Belgrade, analyzing 90 patients diagnosed with IIMs per the 2017 EULAR/ACR criteria. Patients were categorized into dermatomyositis (DM) (n = 37), polymyositis (PM) (n = 35), amyopathic dermatomyositis (ADM) (n = 13), and juvenile dermatomyositis (JDM) (n = 5). A control group of 35 patients with primary Raynaud’s phenomenon was also included. NFC findings, clinical manifestations, and laboratory data were compared across the groups. **Results:** In DM, 81.9% exhibited a scleroderma capillaroscopic pattern, which was also present in 76.9% of ADM patients. In PM, the most common pattern was nonspecific changes (48.6%). JDM patients showed a high prevalence of scleroderma changes (n = 4 (80%)). Scleroderma patterns correlated with Gottron’s papules, heliotrope rash, periungual erythema, Raynaud’s phenomenon, and interstitial lung disease (ILD). No significant differences were found in laboratory parameters across capillaroscopic groups, except for a higher prevalence of anti-Jo1 antibodies in patients with nonspecific capillaroscopic changes. **Conclusions:** NFC is a valuable tool for differentiating IIM subtypes and correlating clinical manifestations with specific capillaroscopic patterns. The high prevalence of scleroderma changes in DM and ADM suggests their potential as a diagnostic and prognostic marker in IIMs. Further research with larger cohorts is warranted to validate these findings.

## 1. Introduction

Idiopathic inflammatory myopathies (IIMs) are rare autoimmune disorders characterized by symmetric, progressive proximal muscle weakness, which can range from mild to severe, potentially leading to patients being bedridden. These disorders can also present with extra-muscular symptoms. Traditionally, IIMs are classified into dermatomyositis (DM), polymyositis (PM), and inclusion body myositis (IBM) [1]. However, recent discoveries of new antibodies and distinct phenotypes, such as antisynthetase syndrome and immune-mediated necrotizing myopathy (IMNM), have led to the development of new classification systems, though these remain unvalidated [2]. In 2017, the European Alliance of Associations for Rheumatology (EULAR) and the American College of Rheumatology (ACR) introduced criteria for the classification of IIMs based on 16 variables divided into six categories (age, muscle weakness, skin manifestations, other clinical manifestations, laboratory measurements, and muscle biopsy); each category carries a specific score indicating the likelihood of an IIM diagnosis. The score can be calculated online. Based on the total score, the EULAR/ACR established the probability levels for diagnosing IIMs. They also made a further sub-classification of IIMs into subgroups, including PM (which includes IMNM), DM (amyopathic dermatomyositis (ADM) and classic), juvenile dermatomyositis (JDM), and IBM [3].

Nailfold capillaroscopy (NFC) is a non-invasive technique which is widely used in rheumatology to assess peripheral microcirculation. It is particularly useful for differentiating primary and secondary Raynaud’s phenomenon and for diagnosing and monitoring systemic sclerosis. The Scleroderma Clinical Trials Consortium and the EULAR Study Group on Microcirculation in Rheumatic Diseases have standardized the descriptions of capillaroscopic findings. Normal NFC findings include capillaries with a diameter of less than 20 µm, a density of more than 7 per mm, and the absence of abnormal morphology or microhemorrhages. Nonspecific changes include reduced density, capillary size between 20 and 50 µm, abnormal morphology, or the presence of microhemorrhages. Scleroderma patterns are categorized into early, active, and late stages based on capillary density, the presence of megacapillaries, and morphological changes [4].

The early type of changes is characterized by the presence of a few enlarged capillary loops or microhemorrhages, with a relatively preserved capillary arrangement and no avascular areas. The active type of changes is marked by a greater number of enlarged capillary loops and megacapillaries, more frequent microhemorrhages, mild-to-moderate capillary loss (small-to-medium avascular areas), and a mildly-to-moderately irregular loop arrangement. The late type of changes is distinguished by a small number of irregularly enlarged capillary loops, absence of microhemorrhages, megacapillaries, large avascular areas, fields with significant capillary loss, known as “deserted areas”, and an irregular loop arrangement with varying morphological shapes and bizarre appearances [5].

NFC is used for both clinical and research purposes and is particularly useful in differentiating primary and secondary Raynaud’s phenomenon, as well as in diagnosing and monitoring systemic sclerosis. Since 2013, abnormal capillaroscopic findings have been a classification criterion for systemic sclerosis and are also used to assess the risk of organ involvement in systemic sclerosis [6].

Capillary changes in DM are similar to those seen in systemic sclerosis, which is why many authors consider DM to be part of the scleroderma spectrum and classify these changes according to Maricq and Cutolo [5,7]. Other authors classify them as “scleroderma-like” changes. Microhemorrhages and giant capillaries are often present. A typical finding is the disrupted arrangement of capillary loops, with areas of neoangiogenesis and the presence of bushy and branched blood vessels [8].

NFC has also shown potential as a biomarker for monitoring disease activity and therapeutic response in IIMs. Capillary loss correlates with disease activity, while hemorrhages are not linked to skin involvement [9,10]. Studies indicate that capillary abnormalities can improve with reduced disease activity and after therapy, particularly in adult patients with dermatomyositis [11]. Differences in capillaroscopic findings among IIM subgroups may enhance differential diagnosis.

This study evaluates the clinical, laboratory, and NFC changes in IIM patients using the EULAR/ACR classification, comparing them to a control group with primary Raynaud’s phenomenon. It also assesses associations between NFC patterns and clinical and laboratory manifestations.

## 2. Materials and Methods

This observational, non-interventional, cross-sectional study was conducted at the Institute of Rheumatology in Belgrade, Serbia. This study was conducted from February 2023 to July 2024, during which we collected and analyzed data from all eligible patients diagnosed with IIMs at our center. We reviewed the medical records of patients diagnosed with IIMs within the past two years using the electronic infoMEDIS system. Ninety patients met the 2017 EULAR/ACR classification criteria for IIMs with a moderate (>55%) to high (>90%) probability of diagnosis. These patients were categorized into four subgroups: DM (37 patients), PM (35 patients), JDM (5 patients), and ADM (13 patients). No patients had inclusion body myositis. The control group consisted of 35 adult patients with primary Raynaud’s phenomenon. JDM patients were excluded from comparison with the control group due to differing pediatric characteristics.

### 2.1. Inclusion Criteria

- Diagnosed with an IIM per the 2017 EULAR/ACR classification criteria.

### 2.2. Exclusion Criteria

- Overlap syndrome with other autoimmune diseases (e.g., systemic sclerosis, systemic lupus erythematosus (SLE), Sjögren’s syndrome).
- Other causes of myopathy (e.g., endocrine, metabolic, infectious, neuropathic, or drug-induced).
- Absence of NFC.

Demographic, clinical, laboratory, and immunological data, as well as NFC findings, were extracted from medical records. Data collected included muscle weakness, fever, Gottron’s sign (erythematous or violaceous macules or plaques overlying the elbows and/or knees), heliotrope rash, periungual erythema, mechanic’s hands, Raynaud’s phenomenon (digital blanching and cyanosis after exposure to cold of the fingers or toes due to vasospasm of the digital arteries and subsequent dilation of capillaries and venules during the ischemic phase), arthritis, arthralgia, smoking status, and interstitial lung disease (ILD). Laboratory values recorded included creatine kinase (CK), aspartate amino transaminase (AST), alanine aminotransferase (ALT), and C-reactive reactive protein (CRP). Immunological markers included ANA titer and pattern, Anti-Jo-1 antibody, and myositis-specific antibodies. Patients with anti-PmScl or anti-Ku antibodies were excluded due to the potential for overlap syndrome.

NFC data included mean capillary density, loop morphology, loop size (normal, enlarged, megacapillaries), microhemorrhages (rare: 2–5; frequent: >6), subpapillary venous plexus, and overall capillaroscopy pattern (normal, nonspecific changes, or scleroderma pattern).

This study adhered to Good Clinical Practice guidelines and the Declaration of Helsinki and was approved by the Ethics Committee of the Institute of Rheumatology (approval no. 18/8, 24 February 2023).

Statistical analyses included parametric (*t*-test) and non-parametric tests (Mann–Whitney U, chi-square, Fisher’s exact test, Kruskal–Wallis). Data were processed using SPSS 29.0 and R 3.4.2.

## 3. Results

A total of 168 patient medical records were reviewed. Ten patients did not meet the inclusion criteria, and sixty-eight patients were excluded due to incomplete medical documentation (missing NFC results) or the presence of concurrent autoimmune diseases. Ultimately, 90 patients met the inclusion and exclusion criteria.

The average age was approximately 51 years for the DM, PM, and ADM groups, while the JDM group had an average age of 10.4 years. Female patients were more prevalent across all groups, ranging from 60% in the JDM group to 80% in the PM group.

### 3.1. Clinical and Laboratory Findings

The most common clinical manifestations among patients with DM and JDM were muscle weakness, Raynaud’s phenomenon, Gottron’s sign/papules, and heliotrope rash. In contrast to JDM patients, DM patients also frequently exhibited arthralgia.

For patients with PM, the most common clinical features included muscle weakness, elevated body temperature, arthralgia, and arthritis.

Patients with ADM most commonly presented with Raynaud’s phenomenon, Gottron’s papules, ILD, elevated body temperature, and arthralgia. Nearly half of the patients with DM, PM, and ADM had positive ANA results, as did 40% of patients with JDM. Among specific antibodies, anti-Jo-1 was the most prevalent. The clinical and immunology findings are summarized in Table 1.

### 3.2. Capillaroscopic Findings

In our cross-sectional study, patients with DM showed a statistically significant decrease in mean capillary density, abnormal morphology, presence of microhemorrhages, and enlarged capillary loops compared to the control group. Specifically, only 2.7% had normal capillaroscopic findings, 16.2% had nonspecific changes, and 81.9% had a scleroderma pattern, which was significantly different from the control group using the chi-square test *p* < 0.001. Within the scleroderma pattern, 32.4% had early changes, 40.5% had active changes, and 8.1% had late changes.

For patients with PM, we found a statistically significant difference in capillary density, abnormal morphology, and the presence of subpapillary venous plexus compared to controls. There were no significant differences in capillary loop width or microhemorrhages. The overall capillaroscopic findings in PM patients showed normal results in 48.6%, nonspecific changes in 48.6%, and a scleroderma pattern in only 2.9%, which was significantly different from the control group as assessed by the chi-square test *p* < 0.001 and Fisher’s exact test. The scleroderma pattern was early.

In patients with ADM, we observed statistically significant reductions in capillary density, abnormal morphology, and enlarged capillary loops. However, there was no increased incidence of microhemorrhages. Nonspecific changes were present in 23.1% of ADM patients, while 76.9% exhibited a scleroderma pattern, which was significantly different from the control group as assessed by Fisher’s exact test. Among those with a scleroderma pattern, 15.4% had early changes, 53.8% had active changes, and 7.7% had late changes.

**Figure 1:**
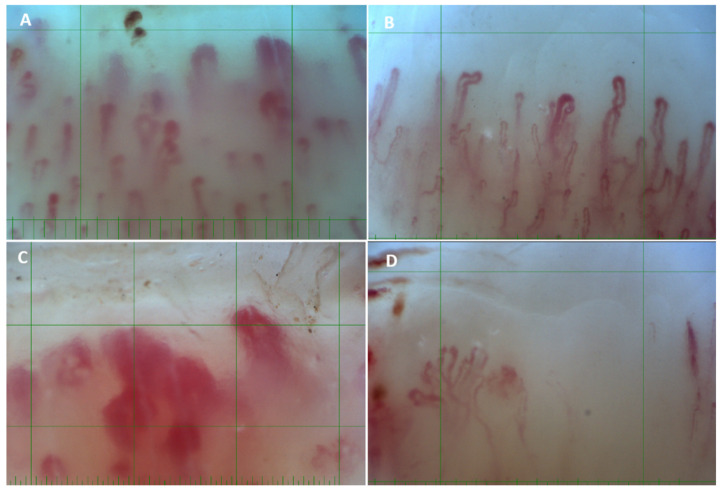
Capillaroscopies of patients with idiopathic inflammatory myopathies at a magnifying power of 250×. (**A**) Patient with dermatomyositis with lower density and the presence of megacapillaries and hemorrhages—active pattern. (**B**) Patients with polymyositis with dilated capillaries but no megacapillaries and reduced density—nonspecific changes. (**C**) Patient with ADM with the presence of megacapillaries and reduced density—active pattern. (**D**) Patient with JDM with very low density, no megacapillaries or microhemorrhages, abnormal morphology, and signs of neoangiogenesis—late pattern.

In JDM patients, a scleroderma pattern was found in four patients, while one patient had nonspecific changes. Among those with scleroderma changes, there were one early, two active, and one late patterns (Table 2 and Table 3, Figure 1).

### 3.3. Association between NFC and Clinical/Laboratory Findings

Based on capillaroscopic findings, patients with IIM were categorized into three groups: normal, nonspecific changes, and scleroderma changes. We examined the association between capillaroscopic findings and clinical and laboratory data, as detailed in Table 4.

There were no statistically significant differences in sex, age, or smoking status among the capillaroscopic groups. However, significant associations were found between capillaroscopic findings and clinical manifestations, including Gottron’s papules, heliotrope rash, periungual erythema, Raynaud’s phenomenon, and interstitial lung disease (ILD). These manifestations were more frequent in patients with scleroderma capillaroscopic changes compared to those with nonspecific changes or normal findings (*p* < 0.001). Conversely, arthralgia and arthritis were more common in the nonspecific changes group (*p* = 0.025; *p* = 0.010).

Muscle weakness and mechanic’s hands approached statistical significance (*p* = 0.056; *p* = 0.057).

No significant differences were found in laboratory parameters such as CK, LDH, AST, ALT, ESR, and CRP among the capillaroscopic groups.

Regarding immunological tests, there was no significant association with ANA positivity (*p* = 0.908). However, a significant association was found with anti-Jo-1 antibodies, which were most prevalent in the nonspecific changes group (*p* = 0.013).

## 4. Discussion

In recent years, NFC has proven to be a valuable diagnostic and prognostic tool in rheumatology, particularly in evaluating microvascular involvement in various autoimmune disorders [12]. This study supports its utility in diagnosing and prognosticating different myositis subtypes by analyzing nailfold capillary patterns.

Our findings align with previous studies by Shenvandan, Manfredi, and Kubo et al. [13,14,15], which reported similar clinical manifestations among patients with DM, though Raynaud’s phenomenon was present in 13.5%, 11.4%, and 11.3% of cases, respectively shown in Table 5. The discrepancy in Raynaud’s phenomenon prevalence may stem from our study’s inclusion criteria, which required completed NFC, typically indicated by the presence of Raynaud’s symptoms. Kubo et al. also noted a higher percentage of ILD at 75.7%, likely due to the inclusion of patients with overlap syndrome [15].

For PM, Manfredi et al. [14] observed a lower prevalence of Raynaud’s phenomenon (21%) and arthritis (6%), but a higher percentage of ILD (64.4%). Shenvandan et al. [13] reported similar findings, with 15% presenting with arthritis and 10% with Raynaud’s phenomenon.

Our NFC findings are consistent with previous studies, except that in their study, Shenvandan et al. [13] examined 19 patients with PM, finding that 42% exhibited a scleroderma capillaroscopic pattern, 47% had nonspecific changes, and 11% had normal findings. However, after excluding patients with overlap syndrome, 11 patients remained with “pure” PM, of whom 63% had nonspecific changes, 27% had normal findings, and 9% exhibited a scleroderma pattern.

In JDM patients, a scleroderma pattern was found in four out of five patients. Interestingly, the “classical scleroderma” pattern, consisting in the simultaneous presence of megacapillaries and avascular areas, was present in 12 out of 17 children with juvenile systemic sclerosis [16].

Comparing NFC findings with the frequency of clinical manifestations revealed a statistically significant association with the presence of Gottron’s papules, heliotrope rash, periungual erythema, Raynaud’s phenomenon, and ILD. These manifestations were more frequently observed in patients with scleroderma changes compared to those with nonspecific changes or normal findings. In contrast, arthralgias and arthritis were more commonly seen in patients with nonspecific capillaroscopic changes. Muscle weakness and mechanic’s hands were on the borderline of statistical significance in terms of frequency. When comparing the mean values of laboratory parameters across different capillaroscopic groups, we found no statistically significant differences in CK, AST, ALT, or CRP levels. Additionally, there was no significant association between ANA positivity and capillaroscopic findings. However, anti-Jo1 antibodies were more frequently detected in patients with nonspecific capillaroscopic changes.

Ganczarczyk et al. demonstrated that avascular zones and enlarged capillaries are associated with Raynaud’s phenomenon, arthritis, and ILD [17]. Mugii et al. found a statistically significant correlation between CK levels and a scleroderma capillaroscopic pattern [10]. Shenvandan identified a significant correlation between the scleroderma capillaroscopic pattern and myopathic findings on EMG [18]. Kubo showed that heliotrope rash, Gottron’s papules or sign, and palmar papules are more frequent in patients with scleroderma capillaroscopic changes. Kubo’s study also revealed that patients with a scleroderma pattern exhibit a higher degree of skin inflammation [15]. In contrast, Sebastiani et al. described scleroderma capillaroscopic changes in 35.3% of patients with antisynthetase syndrome and found a correlation between these changes and the presence of anti-Jo1 antibodies [19].

## 5. Limitation of This Study

This study has certain limitations, primarily due to the design of this research. Specifically, this study was conducted as a cross-sectional study, which means that temporal relationships could not be assessed. Additionally, we only included data from patients who had undergone NFC, which affects the generalizability of the findings.

## 6. Conclusions

NFC offers significant potential as a diagnostic and prognostic tool in the management of IIMs. This study demonstrates that specific capillaroscopic patterns, particularly the scleroderma pattern, are associated with distinct clinical manifestations and laboratory findings in IIM subtypes. While our findings support the use of NFC in the differential diagnosis and prognosis of IIMs, further studies with longitudinal designs are necessary to validate these associations and to establish NFC as a standard diagnostic tool in clinical practice.

## Figures and Tables

**Table 1:** Clinical and immunology finding of patients with idiopathic inflammatory myopathies.

	DM n (%)	PM n (%)	ADM n (%)	JDM n (%)
	37	35	13	5
Age	51.76 ± 12.88	50.46 ± 13.53	51.45 ± 14.64	10.42 ± 3.91
Female	24 (64.9)	28 (80)	10 (76.9)	3 (60)
Muscle weakness	37 (100)	35 (100)	0	4 (80)
Gottron’s sign	27 (73)	0	10 (76.9)	4 (80)
Heliotrope rash	21 (56.8)	0	9 (69.2)	4 (80)
Periungual erythema	13 (35.1)	0	6 (46.2)	3 (60)
Mechanic hand	13 (35.1)	12 (34.3)	2 (15.4)	0
Raynaud’s phenomena	30 (81.1)	14 (40)	10 (76.9)	4 (80)
ILD	14 (37.8)	12 (34.3)	8 (61.5)	0
Arthralgia	21 (56.8)	24 (68.6)	7 (53.8)	3 (60)
Arthritis	12 (32.4)	15 (42.9)	4 (30.8)	2 (40)
ANA	18 (48.6%)	17 (51.4)	6 (46.2)	2 (40)
Anti Jo1	12 (32.4%)	16 (45.7)	4 (30.8)	1 (20)
Myositis blot	15/23 (Mi2 3, PL7 2, PL12 4, Ro52 4, U1RNP 2)	15/21, PL7 5, PL12 2, EJ 1, Ro52 7	5/12 (PL7 1, Pl12 1, Mi2 1, Ro52 1, MDA5 1)	0

ILD—interstitial lung disease, DM—dermatomyositis, PM—polymyositis, ADM—amyopathic dermatomyositis, JDM—juvenile dermatomyositis.

**Table 2:** Nailfold capillaroscopy findings patients with idiopathic inflammatory myopathies and control group.

Feature	Variable	CON.	DM		PM		ADM		JDM
		N (%)	N (%)	* *p*	N (%)	* *p*	N (%)	* *p*	N (%)
Mean capillary density	Reduced	1 (2.8)	23 (62.1)	<0.001	12 (34.3)	0.006	11 (84.6)	<0.001	2 (40)
	Sign. reduced	0	3(8.1)		1 (2.8)		1 (7.7)		2 (40)
Morphology	Abnormal	5 (14.3)	31 (83.8)	<0.001	16 (45.7)	0.008	12 (92.3)	<0.001	5 (100)
Dimension	Enlarged	1 (2.8)	28 (75.7)	<0.001	1 (2.8)	0.421	9 (69.2)	<0.001	4 (80)
	Giant	0	25 (67.6)		1 (2.8)		9 (69.2)		1 (20)
Micro- hemorr.	Rare	4 (11.4)	19 (51.4)	<0.001	5 (14.3)	0.721	7 (53.8)	0.03	4 (80)
	Frequent	0	7 (18.9)		0		0		0
SBVP	Present	1 (2.8)	3 (8.1)	0.325	18 (51.4)	<0.001	0	0.781	2 (40)
NFC pattern	Normal	32 (91.4)	1 (2.7)	<0.001	17 (48.6)	<0.001	0	<0.001	0
	Nonspecific	3 (8.6)	6 (16.2)		17 (48.6)		3 (23.1)		1
	SD pattern	0	30 (81.1)		1 (2.9)		10 (76.9)		4

CON.—control group, DM—dermatomyositis, PM—polymyositis, ADM—amyopathic dermatomyositis, JDM—juvenile dermatomyositis, Micro-hemorr—microhemorrhages, * *p*—*p* values refer to statistical differences with the control group.

**Table 3:** Scleroderma patterns in patients with idiopathic inflammatory myopathies.

	DM n (%)	PM	ADM	JDM
Early pattern	12 (32.4%)	1 (2.9%)	2 (15.4%)	1 (20%)
Active pattern	15 (40.5%)	0	7 (53.8%)	2 (40%)
Late pattern	3 (8.1%)	0	1 (7.7%)	1 (20%)

DM—dermatomyositis, PM—polymyositis, ADM—amyopathic dermatomyositis, JDM—juvenile dermatomyositis.

**Table 4:** Association between clinical and laboratory findings and capillaroscopy patterns in all patients with idiopathic inflammatory myopathies.

	Capillaroscopy Pattern						*p* Value
	Normal		Nonspecific Pattern		Scleroderma Pattern		
	N	%	N	%	N	%	
Sex (F)	15	83.3%	19	70.4%	30	66.7%	0.417
Smoking	8	44.4%	9	33.3%	16	35.6%	0.733
Muscle weakness	18	100.0%	24	88.9%	35	77.8%	0.056
Gottron’s sign	0	0.0%	5	18.5%	36	80.0%	<0.001
Heliotrope rash	1	5.6%	6	22.2%	27	60.0%	<0.001
Periungual erythema	0	0.0%	2	7.4%	20	44.4%	<0.001
Mechanic hand	2	11.1%	12	44.4%	13	28.9%	0.056
Raynaud’s phenomena	6	33.3%	18	66.7%	38	84.4%	<0.001
ILD	1	5.6%	11	40.7%	23	51.1%	0.004
Arthralgia	8	44.4%	22	81.5%	25	55.6%	0.025
Arthritis	2	11.1%	15	55.6%	16	35.6%	0.010
ANA-positive	9	50.0%	14	51.9%	21	46.7%	0.908
Anti Jo1	4	22.2%	16	59.3%	13	28.9%	0.013
CK IU/L AS ± SD	3663.6 ± 3257.8		3570.7 ± 5460		3198 ± 3996		0.336 *
AST IU/L AS ± SD	147.2 ± 95.7		198 ± 337.1		143 ± 181.3		0.286 *
ALT IU/L AS ± SD	128.2 ± 111.1		106.6 ± 122.3		96.7 ± 111.2		0.169 *
CRP mg/L AS ± SD	19.3 ± 15.1		24.0 ± 24.2		21.6 ± 24.6		0.786 *
Age (years) AS ± SD	54.8 ± 13.6		47.2 ± 15.4		47.6 ± 17.0		0.223 *

F—female, ILD—interstitial lung disease, CK—creatine kinase, AST—aspartate amino transaminase, ALT—alanine aminotransferase, CRP—C-reactive reactive protein. *—*p* value determined with Kruskal–Wallis test.

**Table 5:** Literature review on findings of nailfold capillaroscopy in idiopathic myopathies [11,12,13,15,16,17,18,19,20,21].

Authors	N of Patients	Diagnosis	Capillaroscopy Finding
Ganczarczyk et al. [17]	35	PM 19 DM 16	Avascular zones were observed in 94% of patients with DM and 42% of patients with PM. Enlarged capillaries were present in 56% of DM patients and 21% of PM patients.
Mugii et al. [10]	50	DM	Scleroderma capillaroscopic patterns were found in 74% of patients: early in 24%, active in 46%, and late in 4%.
Shenvandan et al. [13]	150	DM 81 ADM 25 JDM 25 PM 19	Among DM patients, 72.8% exhibited a scleroderma capillaroscopic pattern, while 27.16% had nonspecific changes. Among patients with ADM, 76% had a scleroderma capillaroscopic pattern, 16% had nonspecific changes, and 8% had normal findings. In JDM patients, 72% had a scleroderma capillaroscopic pattern and 27% had nonspecific changes. In PM patients, 42% showed a scleroderma capillaroscopic pattern, 47% had nonspecific changes, and 11% had normal findings.
Bergman et al. [20]	11	DM	Overall, 63% of patients had scleroderma capillaroscopic changes, while 36% had normal findings.
Ingegnoli et al. [21]	8	JDM	Scleroderma changes were present in 63% of the subjects, with no patients showing normal capillaroscopic findings.
Nascif et al. [22]	13	JDM	In the active phase, 92% (12/13) of patients had scleroderma capillaroscopic changes.
Pavlov Dolijanovic et al. [23]	26	PM 19 DM 7	Among PM patients, 68.42% had normal findings and 31.57% had nonspecific changes. In DM patients, 71.1% had a scleroderma pattern, while 28.9% had nonspecific changes.
Manfredi et al. [14]	52	DM 29 PM 23	A scleroderma capillaroscopic pattern was observed in 69% of DM patients, while none of the PM patients had a scleroderma pattern.
Shenvandan et al. [18]	27	DM	A scleroderma pattern was present in 88.9% of patients, with an early form in 3%, active form in 33%, and late form in 52%. Nonspecific changes were found in 11%.
Kubo et al. [15]	70	DM 52 PM 18	A scleroderma capillaroscopic pattern was observed in 65.4% of patients with dermatomyositis (DM) and 27.8% of patients with polymyositis (PM).
Our study	90	DM 37 PM 35 ADM 13 JDM 5	DM patients: 81.1% exhibited scleroderma capillaroscopic changes, 16.2% had nonspecific changes, and 2.7% had normal findings. In PM patients: 2.9% had scleroderma capillaroscopic changes, 48.6% had nonspecific changes, and 48.6% had normal findings. For patients with amyopathic dermatomyositis (ADM): 76.9% had scleroderma capillaroscopic changes, while 23.1% had nonspecific changes. In JDM patients: 80% had scleroderma capillaroscopic changes and 20% had nonspecific changes.

DM—dermatomyositis, PM—polymyositis, ADM—amyopathic dermatomyositis, JDM—juvenile dermatomyositis.

## Data Availability

Data are contained within the article.
